# Developing nursing approaches across the chronic illness trajectory: a grounded theory study of care from diagnosis to end-of-life in Western Amhara, Ethiopia

**DOI:** 10.3389/frhs.2025.1502763

**Published:** 2025-06-11

**Authors:** Abebe Dilie Afenigus, Mastewal Ayehu Sinshaw

**Affiliations:** ^1^ Department of Nursing, College of Medicine and Health Sciences, Debre Markos University, Debre Markos, Ethiopia; ^2^ Tibebe Ghion Specialized Hospital, Bahir Dar University, Bahir Dar, Ethiopia

**Keywords:** nursing approaches, chronic illness trajectory, grounded theory, diagnosis, end-of-life

## Abstract

**Background:**

Managing chronic illness requires navigating a complex trajectory from diagnosis to end-of-life, with each phase necessitating specific nursing approaches. Effective management throughout these phases is vital for improving patient outcomes and quality of life.

**Objective:**

This study aims to explore nursing approaches in managing chronic illness across its trajectory, from diagnosis to end-of-life care, focusing on phase-specific care, emotional support, education, interdisciplinary collaboration, and the challenges faced by nurses.

**Methods:**

A qualitative research design using a grounded theory approach was employed to construct a theoretical framework grounded with the insights and experience of nurses’ approaches across the chronic illness trajectory within Western Amhara, Ethiopia. The study comprised 24 nurses who were selected through the process of purposeful and theoretical sampling methods. Data was collected via in-depth interviews. Data analysis followed a constant comparative method, involving open, axial, and selective coding to identify key strategies and challenges across the illness trajectory.

**Results:**

The primary finding of this study emphasizes the evolving and adaptive role of nurses in chronic illness management, highlighting their ability to provide personalized care, emotional support, and education throughout the illness trajectory. Central to the investigation is the theory of nurses' evolving and adaptive role in chronic illness management, where they adjust their strategies to address the physical, emotional, and psychological needs of patients and families, from pre-diagnosis to end-of-life care. The study identifies key adaptive strategies, including fostering resilience, facilitating interdisciplinary collaboration, and managing fluctuating symptoms. Despite challenges such as heavy workloads and emotional strain, nurses require training for continuous professional development, technological integration, and collaborative platforms to reinforce their critical role in optimizing patient outcomes in chronic illness management.

**Conclusion:**

This study highlights nurses' adaptive role in chronic illness care, focusing on phase-specific interventions, emotional support, interdisciplinary collaboration, and education across entire illness trajectory to meet diverse needs of patients and their families. Despite challenges such as heavy workloads and emotional strain, the study recommends ongoing professional development and technological integration to optimize patient outcomes.

## Introduction

Chronic illnesses are among the leading health challenges worldwide, including low and middle income countries (LMICS) like Ethiopia and managing these conditions requires comprehensive, long-term care that spans from diagnosis through to end-of-life (1, 2). The burden and prevalence of chronic diseases like diabetes, hypertension, asthma, cardiovascular diseases, and cancer is rising, making it crucial to develop effective, adaptive, and culturally appropriate nursing approaches to manage these conditions at every stage of the illness trajectory. The care journey for individuals with chronic illnesses is complex and multifaceted, involving several phases: pre-trajectory (before diagnosis), trajectory onset (diagnosis), stable, unstable, acute, crisis, comeback, downward, and dying phases (1, 3, 4). Throughout these stages, nurses play a pivotal role in ensuring that patients receive continuous, individualized, and supportive care (5). However, there is limited research exploring how nursing care is delivered across the full spectrum of the chronic illness trajectory in LMICs, including Ethiopia (6, 7). Nurses must navigate not only clinical challenges but also social, emotional, and psychological dimensions of chronic illness, which demand sensitive and adaptive care strategies (8, 9).

During the pre-trajectory phase, when symptoms are present but a diagnosis has not yet been made, nurses provide essential support through early identification, awareness educational sessions, symptom monitoring, and addressing patients' emotional concerns (10). As the trajectory onset phase, once a diagnosis is made, nurses support patients by offering guidance, education, and developing personalized care plans to facilitate adaptation (11). In the stable phase, nurses work to maintain the patient's health by promoting adherence to treatment plans and fostering lifestyle changes (12). However, as patients enter the unstable phase, nurses must remain flexible, continuously adjusting care strategies to address fluctuating health conditions (13).

The acute and crisis phases represent moments of heightened vulnerability for patients, demanding prompt interventions and multidisciplinary collaboration (14, 15). During the comeback phase, nurses focus on recovery and rehabilitation, supporting patients in regaining strength and adjusting to new health realities (3). As patients transition into the downward phase, characterized by progressive health decline, nurses provide palliative care, focusing on symptom relief, emotional support, and preparation for end-of-life decisions (4). In the dying phase, palliative care becomes central, with nurses offering compassionate end-of-life care, ensuring dignity and comfort for both the patient and their family (16).

Although the role of nurses across these phases is increasingly recognized, there is a need for a deeper understanding of how nursing approaches can be adapted to the varying needs of patients throughout the chronic illness trajectory (17). In Ethiopia, cultural beliefs about illness causation, stigma associated with chronic conditions, and traditional health practices often influence how patients seek care and adhere to treatment, which can pose challenges for nurses (18, 19). It is important to develop nursing strategies that are effective, culturally appropriate, and sustainable to support patients throughout the chronic illness trajectory, especially in the Western Amhara region of Ethiopia.

This grounded theory study aims to explore the strategies employed by nurses and to understand the challenges and solutions across the chronic illness trajectory. The findings will provide valuable insights into how nurses can adapt their care strategies to meet the evolving needs of patients, enhance interdisciplinary collaboration, and improve the overall quality of life for individuals living with chronic illness in Ethiopia. Therefore, this study is significant for improving nursing practice across the chronic illness trajectory and for informing nursing education.

### Objective

• The general objective of this study is to explore and develop a grounded theory of nursing approaches in managing chronic illness across its trajectory, from diagnosis to end-of-life care, with a focus on phase-specific care, emotional support, education, interdisciplinary collaboration, and the challenges faced by nurses in Western Amhara, Ethiopia.

### Research questions

• How do nurses adapt their care approaches to meet the evolving needs of patients across the different phases of the chronic illness trajectory (pre-trajectory, trajectory onset, stable, unstable, acute, crisis, comeback, downward, and dying)?
• How do nurses collaborate with interdisciplinary teams to manage complex chronic illness, and what role does patient education play in adapting care for each phase?
• What nursing interventions and strategies do nurses employ to provide emotional, psychological, and physical support to patients during each phase of chronic illness?
• What are the key challenges nurses face in chronic illness management, and how can nursing practice be improved through enhanced training, monitoring technologies, and interdisciplinary collaboration to optimize patient outcomes?

## Methods

### Study design

This qualitative study utilized the Grounded Theory Method to explore nursing approaches to managing chronic illness across its trajectory, from diagnosis to end-of-life care. Grounded Theory was chosen for its ability to facilitate theory development directly from data, providing a comprehensive and in-depth understanding of nursing practices, challenges, and decision-making processes in chronic illness management. This methodology enables the inclusion of new concepts that may not be captured by pre-existing theoretical frameworks, allowing flexibility and adaptability in interpretation. As such, the study remains rooted in the lived experiences of participants, ensuring that the insights generated reflect the actual complexities of nursing care across the chronic illness trajectory.

The study was guided by the Chronic Illness Trajectory Framework (CITF), developed by Corbin and Strauss, which served as a starting point for exploring nursing approaches in chronic illness management (20, 21). However, the flexibility inherent in grounded theory allows for the inclusion of new, emergent concepts beyond those covered by the CITF, ensuring a more nuanced and context-specific understanding.

The study adheres to the guidelines outlined in the Consolidated Criteria for Reporting Qualitative Research (COREQ) checklist, ensuring rigor and transparency throughout the research process (22).

### Study setting and period

The research was conducted in two hospitals located in Western Amhara, Ethiopia: Finote Selam General Hospital and Tibebe Ghion Specialized Hospital. These hospitals were selected because they serve diverse populations, including individuals in various stages of chronic illness. Data collection took place in the inpatient departments and chronic follow-up units, where nurses provided care to patients with chronic illnesses across different phases of the illness trajectory. The study spanned a period of 6 months, from January 2024 to June 2024, which allowed for extensive data collection and analysis.

### Participants

The participants in the study were nurses with direct experience in managing patients with chronic illnesses across different phases of the illness trajectory. The inclusion criteria for participants required them to have at least 1 year of experience in chronic illness management and to have actively engaged in care across multiple phases, including acute, stable, and crisis stages. Nurses were recruited from healthcare institutions in western Amhara.

### Sampling technique and sample size

The sampling process initially began with purposive sampling and transitioned to theoretical sampling as the study progressed. Participants were chosen based on insights gained from previous participants, continuing until data saturation was achieved when no new or relevant information emerged from additional interviews. This strategy facilitated a thorough exploration and understanding of the topic, drawing on the depth and richness of participants' experiences. Consequently, 24 participants were selected to ensure adequate depth and diversity. As data were gathered, it was analyzed and coded iteratively, with adjustments made to the sample size to guarantee comprehensive coverage of the data.

### Data collection

Data collection began after securing ethical approval. Subsequent interviews were conducted to refine and verify concepts, ensuring the development of a theory grounded in the data. The principal investigator conducted in-depth interviews to explore the experiences, challenges, and strategies used by nurses in managing patients with chronic illnesses. These interviews focused on key aspects such as nursing interventions, patient education, symptom management, emotional support, and interdisciplinary collaboration throughout the chronic illness trajectory. The interview process was flexible, with open-ended questions designed to encourage participants to share their personal experiences, thoughts, and practices. Each face-to-face interview lasted between 50 and 90 min, with an average duration of 60 min, and was recorded with the participants' consent.

The data collection process was iterative, with continuous analysis occurring alongside data gathering. As new themes emerged from the interviews, the researcher adjusted the questions to further explore these emerging topics. This iterative approach allowed for the refinement of the data collection process and ensured that the theory developed was deeply grounded in the participants' real-world experiences, providing a rich and detailed understanding of nursing practices in chronic illness care.

### Data analysis

In this grounded theory study, data were analyzed using an iterative, constant comparative method, aligning with the Corbin and Strauss approach (23). This approach allowed for concurrent data collection and analysis, with each subsequent interview refining the understanding of emerging patterns from previous data.

All interviews were conducted in Amharic, the local language, and were later transcribed and translated into English. Transcripts were read repeatedly to gain a holistic understanding, and meaning units—segments of text expressing a single idea or experience were identified. These meaning units formed the foundation for three interconnected stages of coding: open coding, axial coding, and selective coding.

During the open coding phase, the data were broken down into discrete codes representing actions, interactions, or meanings expressed by participants. These initial codes captured a wide range of nursing activities and experiences, including “emotional support,” “rehabilitation planning,” “care plan adjustment,” and “symptom monitoring.”

In the axial coding phase, related open codes were grouped into categories by examining their relationships and contexts. Categories reflected the evolving nursing roles across specific phases of the chronic illness trajectory such as “Pre-trajectory phase: Navigating uncertainty,” “Stable phase: Maintaining balance,” and “Dying phase: Delivering compassionate end-of-life care.” Other categories emerged around cross-cutting practices such as interdisciplinary collaboration, emotional and psychological support, and patient education.

Finally, selective coding was used to identify the core category that unified the entire dataset:

“The evolving and adaptive role of nurses in chronic illness management.”

This core category encapsulated how nurses continuously adapted their approaches to meet the changing physical, emotional, and psychosocial needs of patients throughout the illness trajectory from diagnosis through to end-of-life care.

Throughout the analysis, attention was given to the context and culture of care delivery, especially in a resource-limited setting like Western Amhara. The relationships among codes, categories, and themes were visually mapped and narratively integrated to develop a theory grounded in participants' experiences. This iterative and rigorous process resulted in a comprehensive and nuanced understanding of how nurses support and empower patients living with chronic illness in Ethiopia.

### Trustworthiness

To enhance the trustworthiness of the study, several strategies aligned with grounded theory methodology were employed. Credibility was achieved through prolonged engagement by spending adequate time in the field and building rapport with participants, which allowed the researcher to gain a deeper understanding of their experiences and behaviors (24). Reflexivity, involving the acknowledgment of personal biases and preconceptions throughout the research process, was also employed to enhance credibility (24). Transferability was supported through thick description, offering rich, detailed accounts of the study context, participants, and methodology (24). Dependability was ensured by maintaining an audit trail that documented the research process, analytical decisions, and methodological choices (24). Finally, confirmability was strengthened through peer debriefings, which helped review interpretations and findings, as well as member checking, where participants reviewed and confirmed the accuracy of their quotations to help reduce researcher bias (24).

## Results

### Background information

Nurses in the field of chronic illness management have been working in various roles, including clinical practice and patient education. In their current roles, they often oversee multidisciplinary teams that provide comprehensive care to patients with long-term health conditions. The common types of chronic illnesses commonly encountered by nurses include diabetes, hypertension, asthma, chronic obstructive pulmonary disease (COPD), heart diseases, asthma, stroke, HIV/AIDS, Parkinson's diseases and rheumatoid arthritis. Their responsibilities involve not only managing these conditions through medication and lifestyle modifications but also educating patients about self-management strategies and coordinating care between different healthcare providers. This includes developing personalized care plans, monitoring patient progress, and addressing any challenges that arise in chronic illness management (Table 1).

**Table 1:** Demographic characteristics of the participants.

Demographic variable	Response	Frequency
Age	20–30 years	8
	31–40 years	10
	≥41 years	6
Gender	Male	9
	Female	15
Educational level	Diploma	8
	Bachelor's degree	12
	Master's degree	4
Years of experience	1–5 years	10
	6–10 years	9
	≥11 years	5
Working unit	Medical ward (inpatient)	18
	OPD (chronic follow up)	6
Work setting	Finote Selam General Hospital	12
	Tibebe Ghion Specialized Hospital	12
Role of nurse	Clinically working nurse	22
	Nurse manager	2

### Themes identified

The analysis of the interviews revealed several core themes that reflect the dynamic roles and responsibilities of nurses as they navigate the chronic illness trajectory. These themes include phase-specific adaptations in care delivery, interdisciplinary collaboration, emotional and psychological support, patient education and empowerment, challenges and solutions in nursing practice, and recommendations for future improvements in nursing practices (Table 2). The central concept that emerged from this grounded theory study is that nurses play a dynamic, adaptive, and supportive role throughout the chronic illness trajectory, with their roles evolving as patients move from diagnosis to end-of-life care.

**Table 2:** Illustration of open codes, categories and themes for the study.

Participant quote/meaning units	Open code	Category	Theme
“In this initial phase, before a diagnosis was made, nurses played a key role in providing emotional support and managing uncertainty. Nurses educated patients about potential symptoms and guided them in tracking their condition while awaiting a diagnosis.”	Emotional support, symptom education, care guidance	Pre-trajectory phase: navigating uncertainty	Phase-specific adaptations in care delivery
“After diagnosis, compassionate support was given, with clear explanations of the condition and treatment options. A personalized care plan was created, linking the patient to resources, support groups, and setting achievable health goals.”	Compassionate support, care planning, linkage	Trajectory onset: adapting to a new diagnosis	Phase-specific adaptations in care delivery.
“In the stable phase, progress was monitored, treatment adherence supported, and the care plan personalized to daily life. Coordination with providers and continuous education on managing the condition and lifestyle changes were emphasized.”	Progress monitoring, treatment adherence, education	Stable phase: maintaining balance	Phase-specific adaptations in care delivery.
“In the unstable phase, frequent assessments were made, care plans adjusted, and additional resources engaged. The patient was guided in tracking symptoms, managing treatment changes, and knowing when to seek help.”	Dynamic planning, symptom tracking	Unstable phase: responding to fluctuations	Phase-specific adaptations in care delivery.
“During the acute episode, immediate interventions were provided, with ongoing monitoring and care adjustments. Clear communication was maintained with the patient and family, and emergency treatments were administered in coordination with the healthcare team, along with emotional support.”	Immediate interventions, care adjustments, support	Acute phase: managing crisis	Phase-specific adaptations in care delivery.
“During the crisis, protocols were quickly implemented, and the interdisciplinary team ensured comprehensive care. The family received clear explanations, and all available resources were used to manage the situation effectively.”	Protocol implementation, crisis coordination	Crisis phase: implementing crisis protocols	Phase-specific adaptations in care delivery.
“In the comeback phase, a personalized rehabilitation plan was created, with ongoing support, physical therapy, counseling, and education on self-management to help the patient adjust and maintain health.”	Rehabilitation plan, ongoing support, self-management education	Comeback phase: supporting recovery	Phase-specific adaptations in care delivery.
“As the patient's health declined, comfort care was prioritized, and the care plan adjusted to meet changing needs. Open dialogue with the family supported adaptation to new limitations and provided emotional support.”	Comfort care, plan adjustment, family dialogue	Downward phase: providing comfort care	Phase-specific adaptations in care delivery.
“In the dying phase, care focused on comfort, pain management, and emotional support. The plan was personalized to the patient's preferences, with family support and guidance on end-of-life issues.”	Pain management, end-of-life support	Dying phase: end-of-life care	Phase-specific adaptations in care delivery.
“Nurses ensured regular communication with healthcare providers, participating in team meetings to ensure coordinated care across the patient's illness trajectory.”	Interdisciplinary communication	Communication with healthcare providers	Interdisciplinary collaboration
“Nurses engaged with community health workers, fostering collaboration that bridged formal healthcare systems.”	Community collaboration	Community-based care integration	Interdisciplinary collaboration
“Teamwork was strengthened by clear role definitions and mutual respect, ensuring coordinated care that addressed all aspects of the patient's needs.”	Team building, respect, role clarity	Team collaboration	Interdisciplinary collaboration
“Nurses established a therapeutic nurse-patient relationship, providing emotional support to help patients manage anxiety, fear, and distress.”	Emotional reassurance, relationship building	Emotional support for patients	Emotional and psychological support
“Nurses offered counseling to families and educated them on how to cope with the emotional strain and assist in caregiving responsibilities.”	Family counseling, stress support	Family support	Emotional and psychological support
“Nurses facilitated connections to grief counseling and support groups, ensuring continued emotional and psychological support.”	Grief counseling, group support	Counseling service facilitation	Emotional and psychological support
“Patients were educated on their condition, treatment plans, and lifestyle changes, with resources provided to enhance understanding.”	Patient education, treatment literacy	Patient education	Education and empowerment
“Self-management strategies were emphasized, with nurses guiding patients in tracking symptoms and making informed decisions about their health.”	Self-monitoring, shared decision-making	Self-management empowerment	Education and empowerment
“Families were provided with education on caregiving techniques and stress management to ensure they could provide effective support.”	Caregiver support, stress education	Family education	Education and empowerment
“Real-time symptom monitoring tools were utilized to track patient progress and adjust care plans swiftly.”	Symptom tracking, care adjustment	Managing fluctuating symptoms	Challenges and solutions in practice
“Emotional resilience training for nurses helped reduce burnout, ensuring sustained compassion and quality care.”	Emotional resilience, burnout prevention	Emotional and psychological stress	Challenges and solutions in practice
“Continuous education in chronic illness management and cultural competence was recommended to enhance nurses’ effectiveness.”	Ongoing training, skill development	Training and professional development	Future improvements in chronic illness management
“Technological advancements, such as real-time symptom tracking, were highlighted as vital for improving care delivery.”	Digital tools, innovation	Technological support for care	Future improvements in chronic illness management
“Building interdisciplinary collaboration platforms will strengthen teamwork and streamline decision-making for improved patient outcomes.”	Collaboration platforms, decision-making	Interdisciplinary care planning	Future improvements in chronic illness management

### Core concept: nurses' adaptive and supportive role in chronic illness management

The central finding of this study is that nurses serve as adaptive and supportive caregivers across various phases of chronic illness. Their role evolves in response to the changing needs of patients, from the initial diagnosis to end-of-life care. Nurses provide multifaceted care that includes emotional support, patient education, care coordination, and symptom management. Their ability to remain flexible in their approach to care, while offering consistent emotional support and guidance, is crucial in managing chronic illness and enhancing patient outcomes.

### Categories and subcategories

1) Phase-specific adaptations in care delivery

Nurses' roles were found to be highly dynamic, evolving as patients progressed through the various phases of chronic illness. Each phase required unique strategies to support patients' changing physical, emotional, and psychological needs.

• Pre-trajectory phase: navigating uncertainty: nurses provided emotional reassurance, symptom education, and guidance as patients awaited diagnosis. *“Emotional support, symptom education, and care coordination were provided, along with reassurance and timely information. The patient was also referred to counseling services and encouraged to track symptoms while awaiting the diagnosis.” (P1)*
• Trajectory onset: adapting to a new diagnosis: Nurses developed personalized care plans and supported patients emotionally during the initial adjustment. *“After diagnosis, compassionate support was given, with clear explanations of the condition and treatment options. A personalized care plan was created, linking the patient to resources, support groups, and setting achievable health goals.” (P4)*
• Stable phase: maintaining balance: nurses emphasized monitoring, adherence to care plans, and life style education. *“In the stable phase, progress was monitored, treatment adherence supported, and the care plan personalized to daily life. Coordination with providers and continuous education on managing the condition and lifestyle changes were emphasized.” (P6)*
• Unstable phase: responding to fluctuations: nurses adjusted care plans dynamically to address health changes. *“In the unstable phase, frequent assessments were made, care plans adjusted, and additional resources engaged. The patient was guided in tracking symptoms, managing treatment changes, and knowing when to seek help.” (P5)*
• Acute phase: managing exacerbations: During the acute phase, nurses responded promptly to sudden health declines by providing immediate clinical interventions, closely monitoring the patient, and coordinating with the healthcare team. They also maintained clear communication with the patient and family, offering reassurance and emotional support throughout the emergency. *“During the acute episode, immediate interventions were provided, with ongoing monitoring and care adjustments. Clear communication was maintained with the patient and family, and emergency treatments were administered in coordination with the healthcare team, along with emotional support.” (P2)*
• Crisis phase: Implementing crisis protocols: In this high-stakes phase, nurses rapidly implemented crisis management protocols, collaborated with interdisciplinary teams, and offered emotional support to both patients and families. Example: *“During the crisis, protocols were quickly implemented, and the interdisciplinary team ensured comprehensive care. The family received clear explanations, and all available resources were used to manage the situation effectively.” (P7)*
• Comeback phase: supporting recovery: nurses provided rehabilitation support and education to encourage self-management. *“In the comeback phase, a personalized rehabilitation plan was created, with ongoing support, physical therapy, counseling, and education on self-management to help the patient adjust and maintain health.” (P8)*
• Downward phase: providing comfort care: Nurses focused on comfort, symptom relief, and emotional support for patients and families. *“As the patient's health declined, comfort care was prioritized, and the care plan adjusted to meet changing needs. Open dialogue with the family supported adaptation to new limitations and provided emotional support.” (P5)*
• Dying phase: Delivering compassionate end-of-life care: In the final phase, nurses provided palliative care focused on pain management, comfort, and emotional support, facilitating a peaceful transition for the patient. Example: *“In the dying phase, care focused on comfort, pain management, and emotional support. The plan was personalized to the patient's preferences, with family support and guidance on end-of-life issues.” (P6)*

2) Interdisciplinary collaboration

Collaboration with other healthcare professionals was a central theme across all phases. Nurses emphasized the importance of regular communication and coordinated care planning to provide holistic and comprehensive care.

• Team communication and coordination

Effective communication among healthcare providers was essential for high-quality care. Nurses actively participated in interdisciplinary meetings and shared patient updates to ensure all healthcare professionals were aligned on care plans. *“Nurses ensured regular communication with healthcare providers, participating in team meetings to ensure coordinated care across the patient's illness trajectory.” (P1)*

• Collaborating with community-based healthcare providers

Nurses recognized the importance of integrating traditional healers and community-based healthcare providers into the care team. They worked to combine cultural beliefs with modern healthcare practices, ensuring continuous and culturally sensitive care. *“Nurses engaged with community health workers, fostering collaboration that bridged formal healthcare systems.” (P9)*

• Multidisciplinary care team building

A strong, cooperative multidisciplinary team was critical for delivering effective patient care. Nurses worked to define roles clearly, engage in joint decision-making, and ensure mutual respect among all team members. *“Teamwork was strengthened by clear role definitions and mutual respect, ensuring coordinated care that addressed all aspects of the patient's needs.” (P15)*

3) Emotional and psychological support

Emotional support emerged as a cornerstone of nursing practice, with nurses offering reassurance, facilitating counseling services, and creating a supportive environment for both patients and their families.

• Support for patients

Nurses built therapeutic relationships to alleviate fear and anxiety. *“Nurses established a therapeutic nurse-patient relationship, providing emotional support to help patients manage anxiety, fear, and distress.” (P19)*

• Support for families

Families were counseled and educated on how to provide care and cope emotionally. *“Nurses offered counseling to families and educated them on how to cope with the emotional strain and assist in caregiving responsibilities.” (P3)*

• Facilitating counseling services

Nurses facilitated access to mental health services, encouraging patients and families to seek professional counseling or support groups, particularly during end-of-life care. *“Nurses facilitated connections to grief counseling and support groups, ensuring continued emotional and psychological support.” (P7)*

4) Education and empowerment

Nurses served as educators, empowering patients and families with knowledge about illness management and available resources, fostering active participation in care.

• Patient education

Patient Nurses provided patients with detailed information about their illness, treatment options, and symptom management strategies, empowering them to actively participate in their care. *“Patients were educated on their condition, treatment plans, and lifestyle changes, with resources provided to enhance understanding.” (P14)*

• Self-management empowerment

Nurses emphasized the importance of self-management, encouraging patients to set achievable health goals and develop skills for managing their condition independently. *“Self-management strategies were emphasized, with nurses guiding patients in tracking symptoms and making informed decisions about their health.” (P21)*

• Family education

Nurses educated family members to help them assist in day-to-day care and manage their own emotional well-being during the caregiving process. *“Families were provided with education on caregiving techniques and stress management to ensure they could provide effective support.” (P23)*

5) Challenges and solutions in chronic illness nursing practice

Nurses identified several challenges, including symptom fluctuations, emotional stress, and coordination of care. Solutions involved flexible care plans, improved communication, and the use of technology.

• Managing fluctuating symptoms

Nurses continuously adjusted care plans based on symptom changes, leveraging advanced technologies for real-time monitoring. *“Real-time symptom monitoring tools were utilized to track patient progress and adjust care plans swiftly.” (P6)*

• Emotional and psychological stress

Emotional stress, both for patients and providers, was recognized as a significant challenge. Nurses addressed this through emotional support and strategies to prevent burnout. *“Emotional resilience training for nurses helped reduce burnout, ensuring sustained compassion and quality care.” (P10)*

6) Future improvements in chronic illness management

Several areas for improvement were identified, including enhanced training, technological advancements, and improved interdisciplinary collaboration.

• Training and professional development

Ongoing professional development and specialized training were seen as essential for improving nurses’ ability to manage complex chronic illness cases. *“Continuous education in chronic illness management and cultural competence was recommended to enhance nurses’ effectiveness.” (P12)*

• Technological advancements

The integration of advanced technologies for symptom monitoring and care coordination was identified as key to improving chronic illness management. *“Technological advancements, such as real-time symptom tracking, were highlighted as vital for improving care delivery.” (P14)*

• Collaborative platforms

Developing integrated platforms for healthcare teams to communicate and collaborate more effectively was seen as a critical step in improving chronic illness care. *“Building interdisciplinary collaboration platforms will strengthen teamwork and streamline decision-making for improved patient outcomes.” (P20)*

### Grounded theory: the evolving and adaptive role of nurses in chronic illness management

**Figure 1:**
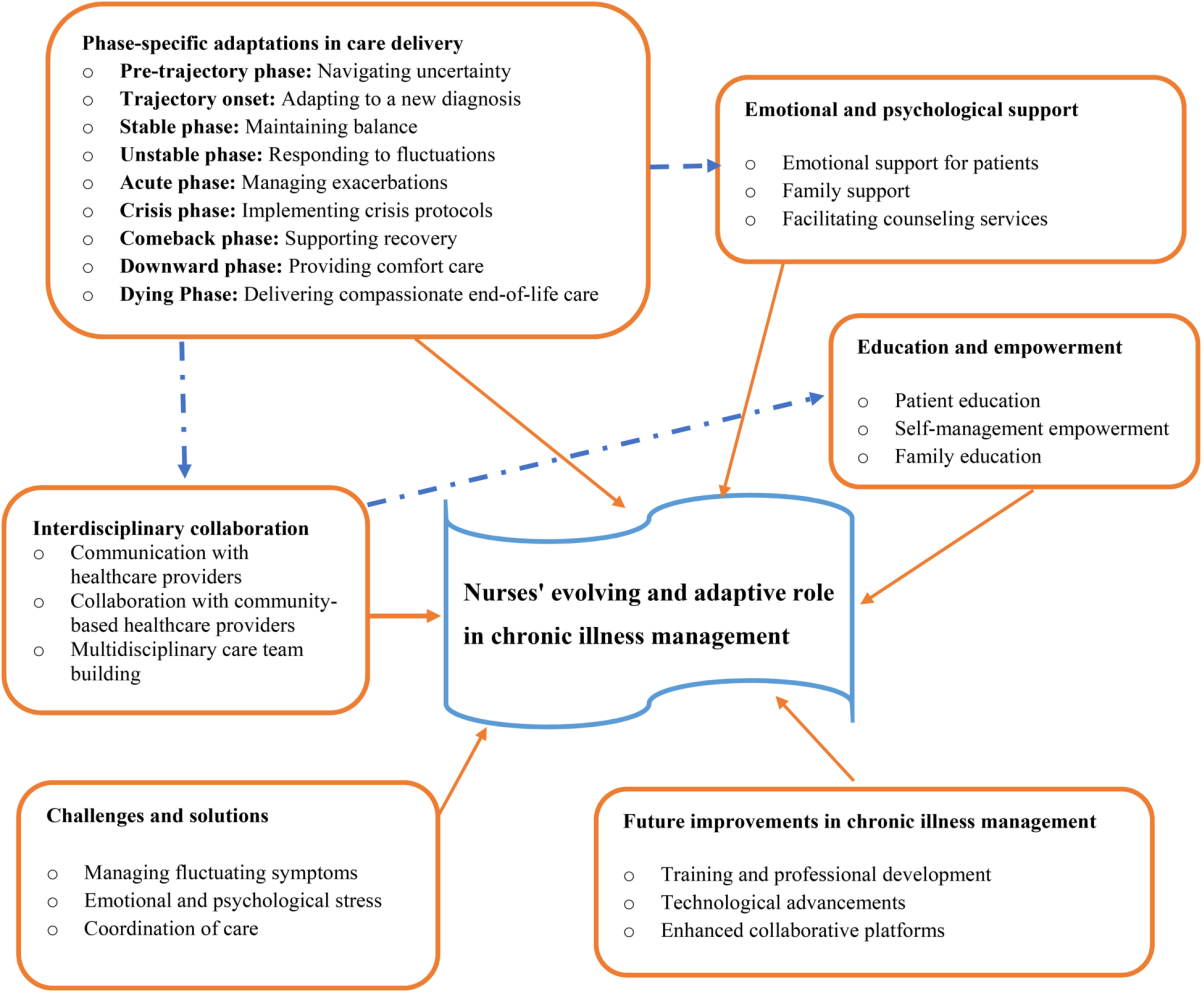
Theory of the evolving and adaptive role of nurses in chronic illness management.

The grounded theory of nurses’ evolving and adaptive role in chronic illness management reveals that nurses are pivotal, dynamic caregivers whose roles transform in response to the changing needs of patients throughout the illness journey. This theory emphasizes that nurses provide a multifaceted, patient-centered approach, offering emotional support, symptom management, education, and care coordination at every stage of the chronic illness trajectory. From the uncertainty of the pre-diagnosis phase to the complexities of end-of-life care, nurses continuously adapt their care strategies to align with the evolving physical, emotional, and psychological needs of the patient and their family (Figure 1).

At the core of the theory is the nurse's ability to offer personalized care and maintain continuity, even as patients' conditions fluctuate. Nurses act as both caregivers and educators, ensuring patients understand their condition and empowering them to take an active role in managing their health. This ongoing education fosters resilience and promotes long-term health management, while also providing emotional support for both patients and families, who often face significant stress. Nurses also facilitate interdisciplinary collaboration, working closely with physicians, specialists, and community providers to ensure a cohesive and coordinated care plan.

Despite challenges such as fluctuating symptoms, emotional strain, and complex care coordination, nurses employ adaptive strategies and resilience training to overcome obstacles and continue providing high-quality care. As chronic illness management evolves, the theory highlights the importance of continuous professional development, the integration of technological advancements, and the creation of improved collaboration platforms to enhance patient outcomes and further solidify nurses' essential role in the care process. Through their adaptable, compassionate, and comprehensive approach, nurses are integral to optimizing both the physical and emotional well-being of patients across the entire chronic illness trajectory.

## Discussion

This study aimed to explore the evolving and adaptive roles of nurses in the management of chronic illness across its various phases. Through grounded theory analysis, the findings reveal that nurses play dynamic, multifaceted roles such as care provider, educator, coordinator, and emotional support provider that shift in response to the patient's condition.

The study found that nurses do not hold a fixed function but rather evolve their roles across different stages of chronic illness. In the pre-trajectory phase, nurses primarily acted as emotional supporters and early identifiers of symptoms, initiating referrals and counseling even before formal diagnosis. This approach aligns with prior research's that emphasizes the importance of proactive support and reassurance to reduce patient anxiety and uncertainty (25). Early counseling and referrals to support groups can significantly alleviate distress (26). At the trajectory onset, their role expanded into that of educators and care planners, providing clarity around diagnosis, treatment options, and goal-setting. This is supported by a literature that show effective education and involvement in care enhance patient understanding and adaptation to chronic illness (27). Similarly, modifying education and support based on individual needs enabled patients and families to become well-informed and prepared for the challenges ahead (28).

In the stable phase, the emphasis of nurses is on maintaining stability through regular monitoring and personalized care plans. This is inline with studies that show nurses focus on maintaining health through regular monitoring, adherence support, and integration of treatment into daily routines, reinforced by patient education strategies to effectively manage chronic diseases (28, 29). As patients enter the unstable phase, nurses face the challenge of managing fluctuating health conditions and increased medical needs. The study's findings align with research indicating that dynamic care plans and symptom tracking to respond swiftly to fluctuating conditions, mobilizing additional resources and modifying treatments as needed (30, 31).

During acute episodes, the role of nurses becomes critical in providing immediate interventions and stabilizing the patient's condition. The findings support existing literatures which show their role includes emphasizing immediate intervention, intensive monitoring, and coordinated emergency care, along with emotional support for patients and families (32, 33). In crisis situations, the need for swift action and crisis management protocols is crucial. This finding is inline with studies that indicate nurses rapidly implement crisis protocols, collaborate with interdisciplinary teams, and ensure clear communication, demonstrating the importance of unified, timely action in high-risk scenarios (34, 35).

Post crisis, the comeback phase focuses on supporting recovery and adjustment. This is congruent with studies that indicate nurses design personalized rehabilitation plans and provide continuous support through physical therapy and psychosocial care, aiding long-term recovery at this phase (36, 37). As patients enter downward and dying phase, the focus shifts to palliative care. This is congruent with other studies that aim to provide components of palliative care at this phases such as symptom management, comfort care, help patient and families and pain relief (38–41).

The study identifies several common challenges faced by nurses in chronic illness management. Emotional strain, complex care coordination, and heavy workload were consistent obstacles in providing optimal care. The findings align with existing literature that highlights the need for flexible care plans and consistent emotional support to address these challenges (42). Improved coordination and communication among healthcare professionals are also critical, reflecting research that emphasizes the importance of interdisciplinary collaboration in managing complex cases (43).

Educating patients and families about the chronic illness trajectory is crucial for effective management. The study's findings confirm that clear, comprehensive education personalized to the patient's understanding enhances their ability to navigate the illness phases (28, 44). Providing written materials, resources, and connecting patients with support groups are effective strategies for improving patient and family knowledge and preparedness, as supported by research on patient education and self-management (45, 46).

Effective collaboration with other healthcare professionals is essential for comprehensive chronic illness care. The study's results highlight the importance of regular communication, coordinated care planning, and interdisciplinary meetings, consistent with literature on the benefits of integrated care approaches (47). Fostering strong working relationships and sharing patient information are key to ensuring all aspects of care are addressed, reflecting best practices in collaborative healthcare (48).

Finally, the study identifies several key areas for improvement in nursing practices, including the need for enhanced professional development, the integration of technological advancements, and the creation of interdisciplinary collaboration platforms. These findings are consistent with studies that suggest staying updated with the latest treatment modalities and utilizing technology for real-time data tracking can significantly improve chronic illness management (49). The establishment of integrated collaboration platforms can facilitate communication and decision-making, thereby improving patient outcomes and optimizing healthcare delivery which is consistent with a study that states promoting effective interdisciplinary collaboration through integrated care platforms is recommended to enhance overall patient care and outcomes (50).

## Strength and limitation of the study

### Strength of the study

• The study is situated in a real-world, high-need context, which enhances its practical relevance.
• It adopts a holistic framework by addressing all stages of the chronic illness trajectory.
• The focus on nursing care and the adaptive strategies employed by nurses in a resource-limited setting is both novel and significant.
• The choice of grounded theory methodology aligns well with the exploratory nature of the research.

### Limitation of the study

• Limited generalizability: as a qualitative study in two hospitals within a single region (Western Amhara), the findings may not be easily transferable to other geographic, cultural, or healthcare settings in Ethiopia or other LMICs. The sample may not capture variability in practices from rural clinics or private facilities.
• Theoretical saturation ambiguity: while theoretical sampling and saturation are mentioned, the process for determining when saturation was achieved (e.g., how redundancy was assessed) could be more explicitly documented to support transparency and rigor.

## Conclusion

This study highlights the evolving and adaptive role of nurses in managing chronic illnesses, underlining their multifaceted approach across various stages of the illness trajectory. Nurses provide not only clinical care but also serve as emotional support providers, educators, and coordinators, demonstrating flexibility and adaptability in responding to the changing needs of patients from the uncertainty of the pre-trajectory phase to the complexities of end-of-life care. Through fostering resilience, facilitating interdisciplinary collaboration, and offering continuous patient education, nurses optimize patient outcomes and ensure comprehensive care. However, challenges such as fluctuating symptoms, emotional strain, heavy workload and the need for effective care coordination remain persistent.

## Data Availability

The original contributions presented in the study are included in the article/Supplementary Material, further inquiries can be directed to the corresponding author.

## References

[1] Rolland JS. Chronic illness and the life cycle: a conceptual framework. Fam Process. (1987) 26(2):203–21. 10.1111/j.1545-5300.1987.00203.x 3595826

[2] World Health Organization. Noncommunicable diseases country profiles 2018. (2018).

[3] Corbin J Strauss A. Managing chronic illness at home: three lines of work. Qual Sociol. (1985) 8(3):224–47. 10.1007/BF00989485

[4] Heitkemper MM Dirksen SR Lewis SM Bucher L Harding M. Medical-Surgical Nursing: Assessment and Management of Clinical Problems. 9th ed. St. Louis, MO: Elsevier (2014). p. 61–4.

[5] Melnyk BM Fineout-Overholt E. Evidence-Based Practice in Nursing & Healthcare: A Guide to Best Practice. Philadelphia, PA: Lippincott Williams & Wilkins (2022).

[6] Toniolo J Ngoungou EB Preux P-M Beloni P. Role and knowledge of nurses in the management of non-communicable diseases in Africa: a scoping review. PLoS One. (2024) 19(4):e0297165. 10.1371/journal.pone.0297165 38635822 PMC11025970

[7] Tamene A. Evidence based assessment of a strategy tailored for prevention and control of chronic illness in selected health centers Addis Ababa, Ethiopia: Purely Qualitative Study Design (2019).

[8] Alruwaili MSZ Maatabi GHA Alatwi NAS Alruwaili EMS Al Ruwaili WSZ Al Ruwaili RFO The role of nursing in improving the quality of life for patients with mental illnesses. Gland Surg. (2024) 9(2):246–52. Available at: http://www.glandsurgery.net/index.php/GS/article/view/84/81

[9] Holmes AM Deb P. The effect of chronic illness on the psychological health of family members. J Ment Health Policy Econ. (2003) 6(1):13–22. Available at: https://pubmed.ncbi.nlm.nih.gov/14578544/14578544

[10] Burton CR. Re-thinking stroke rehabilitation: the Corbin and Strauss chronic illness trajectory framework. J Adv Nurs. (2000) 32(3):595–602. 10.1046/j.1365-2648.2000.01517.x 11012801

[11] Tang X Sun H Ge S Han S Li Y Wu B. Experiences and needs of older adults at different stages of cerebral infarction based on trajectory theory—a qualitative study. Nurs Open. (2023) 10(3):1482–91. 10.1002/nop2.1398 36322506 PMC9912390

[12] Pender NJ Murdaugh CL Parsons MA. Health Promotion in Nursing Practice. London: Pearson Education (2006).

[13] World Health Organization. Transitions of Care: Technical Series on Safer Primary Care. Geneva: World Health Organization (2016).

[14] Nolte E Knai C Saltman R. Assessing chronic disease management in European health systems. In: Nolte E Knai C Saltman RB, editors. Concepts and Approaches. Copenhagen: European Observatory on Health Systems and Policies (2014). p. 1–97. 29019637

[15] Gaskin CJ Ockerby CM Smith TR Russell V O'Connell B. The challenges acute care nurse unit managers face and the strategies they use to address them: perceptions of directors of nursing and nurse unit managers. J Manag Organ. (2012) 18(5):625–40. 10.5172/jmo.2012.18.5.625

[16] Corbin JM Strauss A. Unending Work and Care: Managing Chronic Illness at Home. San Francisco, CA: Jossey-bass (1988).

[17] Craig H Edwards J. Adaptation in chronic illness: an eclectic model for nurses. J Adv Nurs. (1983) 8(5):397–404. 10.1111/j.1365-2648.1983.tb00463.x 6556200

[18] Atinafu D Getaneh G Setotaw G. Assessment of patient safety culture and associated factors among healthcare professionals in public hospitals of Bahir Dar city, northwest Ethiopia: a mixed-methods study. PLoS One. (2024) 19(11):e0313321. 10.1371/journal.pone.0313321 39509370 PMC11542836

[19] Hodes R. Cross-cultural medicine and diverse health beliefs. Ethiopians abroad. West J Med. (1997) 166(1):29–36. Available at: https://pubmed.ncbi.nlm.nih.gov/9074336/9074336 PMC1303953

[20] Reed E Corner J. Defining the illness trajectory of metastatic breast cancer. BMJ Support Palliat Care. (2015) 5(4):358–65. 10.1136/bmjspcare-2012-000415 24644176 PMC4680129

[21] Corbin JM Strauss A. A nursing model for chronic illness management based upon the trajectory framework. Sch Inq Nurs Pract. (1991) 5(3):155–74. 10.1891/0889-7182.5.3.155 1763239

[22] Tong A Sainsbury P Craig J. Consolidated criteria for reporting qualitative research (COREQ): a 32-item checklist for interviews and focus groups. Int J Qual Health Care. (2007) 19(6):349–57. 10.1093/intqhc/mzm042 17872937

[23] Corbin J Strauss A. Basics of Qualitative Research: Techniques and Procedures for Developing Grounded Theory. Thousand Oaks, CA: Sage publications (2014).

[24] Ahmed SK. The pillars of trustworthiness in qualitative research. J Med Surg Public Health. (2024) 2:100051. 10.1016/j.glmedi.2024.100051

[25] Labrague LJ McEnroe-Petitte DM Leocadio MC Van Bogaert P Cummings GG. Stress and ways of coping among nurse managers: an integrative review. J Clin Nurs. (2018) 27(7-8):1346–59. 10.1111/jocn.14165 29148110

[26] Yang M Zhang Z Nice EC Wang C Zhang W Huang C. Psychological intervention to treat distress: an emerging frontier in cancer prevention and therapy. Biochim Biophys Acta Rev Cancer. (2022) 1877(1):188665. 10.1016/j.bbcan.2021.188665 34896258

[27] McCorkle R Ercolano E Lazenby M Schulman-Green D Schilling LS Lorig K Self-management: enabling and empowering patients living with cancer as a chronic illness. CA Cancer J Clin. (2011) 61(1):50–62. 10.3322/caac.20093 21205833 PMC3058905

[28] McGowan PT. Self-management education and support in chronic disease management. Prim Care. (2012) 39(2):307–25. 10.1016/j.pop.2012.03.005 22608868

[29] Burnier M. The role of adherence in patients with chronic diseases. Eur J Intern Med. (2024) 119:1–5. 10.1016/j.ejim.2023.07.008 37479633

[30] Goodwin N Sonola L Thiel V Kodner D. Co-Ordinated Care for People With Complex Chronic Conditions. Key Lessons and Markers for Success. London: The King's Fund (2013).

[31] Lavori PW Dawson R. Adaptive treatment strategies in chronic disease. Annu Rev Med. (2008) 59(1):443–53. 10.1146/annurev.med.59.062606.122232 17914924 PMC2739674

[32] Pines JM Lotrecchiano GR Zocchi MS Lazar D Leedekerken JB Margolis GS A conceptual model for episodes of acute, unscheduled care. Ann Emerg Med. (2016) 68(4):484–91.e3. 10.1016/j.annemergmed.2016.05.029 27397857

[33] Foo CD Yan JY Chan ASL Yap JC. Identifying key themes of care coordination for patients with chronic conditions in Singapore: a scoping review. Healthcare. (2023) 11(11):1546. 10.3390/healthcare11111546 37297686 PMC10252872

[34] Clarke JL Bourn S Skoufalos A Beck EH Castillo DJ. An innovative approach to health care delivery for patients with chronic conditions. Popul Health Manag. (2017) 20(1):23–30. 10.1089/pop.2016.0076 27563751 PMC5278805

[35] Holman HR. The relation of the chronic disease epidemic to the health care crisis. ACR Open Rheumatol. (2020) 2(3):167–73. 10.1002/acr2.11114 32073759 PMC7077778

[36] Dekker J de Groot V. Psychological adjustment to chronic disease and rehabilitation–an exploration. Disabil Rehabil. (2018) 40(1):116–20. 10.1080/09638288.2016.1247469 27830936

[37] Jiakponna EC Agbomola JO Ipede O Karakitie L Ogunsina A Adebayo K. Psychosocial factors in chronic disease management: implications for health psychology. Int J Sci Res Arch. (2024) 12(2):117–28. 10.30574/ijsra.2024.12.2.1219

[38] Glasgow RE Huebschmann AG Krist AH Degruy FV. An adaptive, contextual, technology-aided support (ACTS) system for chronic illness self-management. Milbank Q. (2019) 97(3):669–91. 10.1111/1468-0009.12412 31424137 PMC6739607

[39] Dwarswaard J Bakker EJ van Staa A Boeije HR. Self-management support from the perspective of patients with a chronic condition: a thematic synthesis of qualitative studies. Health Expect. (2016) 19(2):194–208. 10.1111/hex.12346 25619975 PMC5055271

[40] Rawlings D Devery K Poole N. Improving quality in hospital end-of-life care: honest communication, compassion and empathy. BMJ Open Qual.. (2019) 8(2):e000669. 10.1136/bmjoq-2019-000669 31259290 PMC6567943

[41] Sandsdalen T Hov R Høye S Rystedt I Wilde-Larsson B. Patients’ preferences in palliative care: a systematic mixed studies review. Palliat Med. (2015) 29(5):399–419. 10.1177/0269216314557882 25680380

[42] De Ridder D Geenen R Kuijer R Van Middendorp H. Psychological adjustment to chronic disease. Lancet. (2008) 372(9634):246–55. 10.1016/S0140-6736(08)61078-8 18640461

[43] Acosta-García H Alfaro-Lara ER Sánchez-Fidalgo S Sevilla-Sánchez D Delgado-Silveira E Juanes-Borrego A Intervention effectiveness by pharmacists integrated within an interdisciplinary health team on chronic complex patients. Eur J Public Health. (2020) 30(5):886–99. 10.1093/eurpub/ckz224 32052027

[44] Adams RJ. Improving health outcomes with better patient understanding and education. Risk Manag Healthc Policy. (2010) 26:61–72. 10.2147/RMHP.S7500 PMC3270921 22312219

[45] Novak M Costantini L Schneider S Beanlands H. Approaches to self-management in chronic illness. Semin Dial. (2013) 26(2):188–94. 10.1111/sdi.12080 23520989

[46] Grey M Knafl K McCorkle R. A framework for the study of self-and family management of chronic conditions. Nurs Outlook. (2006) 54(5):278–86. 10.1016/j.outlook.2006.06.004 17027605

[47] Southerland JH Webster-Cyriaque J Bednarsh H Mouton CP. Interprofessional collaborative practice models in chronic disease management. Dental Clinics. (2016) 60(4):789–809. 10.1016/j.cden.2016.05.001 27671954

[48] JCIPE Update. Collaborative healthcare: interprofessional practice, education and evaluation (JCIPE). JCIPE. (2021) 11(2):1. Available at: https://jdc.jefferson.edu/jcipe/vol11/iss2/1

[49] Milani RV Bober RM Lavie CJ. The role of technology in chronic disease care. Prog Cardiovasc Dis. (2016) 58(6):579–83. 10.1016/j.pcad.2016.01.001 26772623

[50] Al Otaibi MJ Alhashash SY Alrasheedi BM Alanazi KS Al Anezi DH Alenazi AH Collaborative care models: enhancing patient safety through interdisciplinary teams. J Namib Stud. (2022) 32:946–56.

