# Supplementary material for: Developing nursing approaches across the chronic illness trajectory: a grounded theory study of care from diagnosis to end-of-life in Western Amhara, Ethiopia
Source: Front Health Serv. 2025 Jun 11;5:1502763. doi: 10.3389/frhs.2025.1502763 (PMC12187775; doi:10.3389/frhs.2025.1502763)
Supplement: Supplementary file 1 [file Datasheet1.docx]

**Interview guide**

**Developing nursing approaches across the chronic illness trajectory: A grounded theory study of care from diagnosis to end-of-life in Western Amhara, Ethiopia**

**Introduction:**

1. **Greeting and Introduction:**

- Thank you for taking the time to participate in this interview. My name is [Your Name], and I am conducting a study to understand nursing approaches in managing chronic illness from diagnosis to end-of-life care in Western Amhara, Ethiopia. Your experiences and insights are invaluable to this research.
- The purpose of this interview is to gather your thoughts and reflections on the care of patients with chronic illness, particularly in the context of the local healthcare system.

1. **Background Information:**
   - Can you briefly describe your age, educational level, facility/unit you work, experience in nursing, particularly in relation to chronic illness management?
   - What types of chronic illnesses are most commonly encountered in your facility?

**Phase-Specific Questions (Grounded Theory Approach):**

The following questions encourage interviewees to reflect on their experiences, beliefs, and perspectives at each phase of the chronic illness trajectory.

1. **Pre-Trajectory:**

- When patients exhibit symptoms but have not yet received a diagnosis, how do you approach care during this uncertain phase?
- What strategies do you use to support patients and families dealing with anxiety, confusion, or fear before a diagnosis is made? Are there any cultural factors that influence coping mechanisms during this phase?

1. **Trajectory Onset:**

- Once a chronic illness diagnosis is confirmed, what are the key nursing interventions you provide to help patients and their families adjust to the new reality?
- From your experience, what emotional or psychological reactions do patients and families commonly have, and how do you address these reactions in culturally appropriate ways?

1. **Stable Phase:**

- What specific strategies do you use to maintain the stability of patients with chronic illnesses in this phase? Can you describe any unique practices in your setting that help patients remain stable long-term?
- How do you tailor care plans to meet the individual needs of patients during the stable phase? Are there local or cultural factors that influence these care plans?

1. **Unstable Phase:**

- In the unstable phase, where patients experience fluctuating health, how do you manage their care? What challenges arise during this phase, and how do you address them?
- Are there any specific techniques or approaches you use to help patients cope with unpredictable symptoms or health fluctuations?

1. **Acute Phase:**
   - When a patient experiences an acute episode of their chronic illness, what are the critical nursing interventions you implement during this time to ensure immediate care and stability?
   - Are there traditional or community-based practices that you integrate with formal medical interventions during an acute episode?
2. **Crisis Phase:**
   - During a health crisis, how do you provide care to ensure patient stability and immediate relief? How do you collaborate with the multidisciplinary team to respond to the crisis?
   - How do families in Western Amhara typically respond during a crisis? How do you provide support to them alongside the patient during this phase?
3. **Comeback Phase:**
   - After a crisis or acute episode, how do you help patients reintegrate into their daily lives? What rehabilitation strategies do you employ to support recovery in this phase?
   - Are there community support systems in place to aid recovery, and how do you collaborate with these systems?
4. **Downward Phase:**
   - As a patient's health declines, what key considerations guide your nursing care? How do you support patients and families in managing this difficult transition?
   - How do you provide culturally sensitive support to help patients and families cope with increasing limitations and loss of functionality in this phase?
5. **Dying Phase:**
   - How do you approach end-of-life care for patients with chronic illness? What palliative care elements do you consider essential to providing comfort during this phase?
   - What support do you offer to families during the dying phase, particularly in the context of local beliefs, customs, and end-of-life practices?

**General Questions (Grounded Theory Approach):**

1. **Challenges and Solutions:**
   - What are the major challenges you face when providing nursing care across the different phases of chronic illness in your community? How have you developed strategies or approaches to overcome these challenges?
   - How do you navigate the intersection of formal healthcare and traditional health practices in the management of chronic illnesses? Can you share specific examples where both systems worked together?
2. **Patient and Family Education:**
   - How do you educate patients and families about the different phases of the chronic illness trajectory? What methods or approaches do you find most effective in ensuring that they are well-informed and prepared for each phase?
   - Are there specific cultural or language barriers you encounter during the education process? How do you overcome these challenges to ensure that patients and families understand their care?
3. **Interdisciplinary Collaboration:**
   - How do you collaborate with other healthcare professionals (e.g., doctors, community health workers, other nurses) to provide comprehensive care across the illness trajectory? What does effective teamwork look like in your setting?
   - Can you describe how community-based healthcare practitioners are integrated into the care process? How does this collaboration enhance patient outcomes?
4. **Future Improvements:**
   - Based on your experiences, are there areas where nursing practices could be improved to better support patients with chronic illnesses throughout the entire trajectory?
   - What suggestions do you have for enhancing the healthcare system in Western Amhara to improve chronic illness management? Are there specific changes that would make care more effective or accessible for patients and families?

**Conclusion:**

1. **Final Thoughts:**
   - Is there anything else you would like to share regarding nursing approaches to chronic illness care in your region? Any insights or recommendations for improving care across the chronic illness trajectory in Western Amhara?
2. **Thank You:**
   - Thank you for your time and for sharing your valuable insights. Your contributions will help inform the development of nursing approaches that can improve chronic illness care in Western Amhara, Ethiopia.
